# Multi-layer Bundling as a New Approach for Determining Multi-scale Correlations Within a High-Dimensional Dataset

**DOI:** 10.1007/s11538-024-01335-8

**Published:** 2024-07-12

**Authors:** Mehran Fazli, Richard Bertram, Deborah A. Striegel

**Affiliations:** 1 Austere environments Consortium for Enhanced Sepsis Outcomes (ACESO), The Henry M. Jackson Foundation for the Advancement of Military Medicine, Inc., 6720A Rockledge Dr, Bethesda, MD 20817 USA; 2 Department of Mathematics, Florida State University, 1017 Academic Way, Tallahassee, FL 32306 USA; 3 Programs in Neuroscience and Molecular Biophysics, Florida State University, 91 Chieftan Way, Tallahassee, FL 32306 USA

**Keywords:** Clustering method, Dimension reduction, Spectral clustering, Biological network, Correlation network analysis

## Abstract

The growing complexity of biological data has spurred the development of innovative computational techniques to extract meaningful information and uncover hidden patterns within vast datasets. Biological networks, such as gene regulatory networks and protein-protein interaction networks, hold critical insights into biological features’ connections and functions. Integrating and analyzing high-dimensional data, particularly in gene expression studies, stands prominent among the challenges in deciphering these networks. Clustering methods play a crucial role in addressing these challenges, with spectral clustering emerging as a potent unsupervised technique considering intrinsic geometric structures. However, spectral clustering’s user-defined cluster number can lead to inconsistent and sometimes orthogonal clustering regimes. We propose the *Multi-layer Bundling (MLB)* method to address this limitation, combining multiple prominent clustering regimes to offer a comprehensive data view. We call the outcome clusters “bundles”. This approach refines clustering outcomes, unravels hierarchical organization, and identifies bridge elements mediating communication between network components. By layering clustering results, MLB provides a global-to-local view of biological feature clusters enabling insights into intricate biological systems. Furthermore, the method enhances bundle network predictions by integrating the *bundle co-cluster matrix* with the affinity matrix. The versatility of MLB extends beyond biological networks, making it applicable to various domains where understanding complex relationships and patterns is needed.

## Introduction

The growing complexity of biological data has necessitated the development of innovative computational techniques to extract meaningful information and uncover hidden patterns within vast datasets. Biological networks, such as gene regulatory networks, protein-protein interaction networks, and metabolic networks, represent powerful representations of the interconnectedness among biological entities and their functional relationships. These networks serve as invaluable tools in understanding the intricate molecular machinery that governs life’s fundamental processes (Panditrao et al. 2022).

One fundamental challenge in deciphering biological networks is integrating and analyzing high-dimensional data, particularly in the context of gene expression studies (Van Dam et al. 2017; Chowdhury et al. 2020). The advent of high-throughput technologies, like microarray and next-generation sequencing, has provided researchers with a wealth of genomic data. However, understanding how individual genes interact, collaborate, or influence each other remains a complex puzzle.

Clustering techniques are the primary step in solving this puzzle. These methods cluster the biological features based on data-driven correlation values or measured distances (Ezugwu et al. 2022; Xu and Tian 2015; D’haeseleer 2005). They include K-means clustering (MacQueen 1967; Lloyd 1982; Hartigan and Wong 1979), hierarchical clustering (Nielsen 2016), spectral clustering (Pothen et al. 1990; Ng et al. 2001; Von Luxburg 2007), affinity propagation (Frey and Dueck 2007), and Density-Based Spatial Clustering of Applications with Noise (DBSCAN) (Ester et al. 1996). In the case of gene expression data, clustering methods enable identification of groups of genes with similar expression patterns. By grouping genes into clusters or co-expression modules based on their expression profiles, gene clustering sheds light on co-regulated gene sets that often participate in shared biological processes (Chowdhury et al. 2020; Van Dam et al. 2017; Sarmah and Bhattacharyya 2021). These clusters offer insights into the coordinated behavior of genes, the activation of specific pathways, and potential regulatory mechanisms (Yang et al. 2014; Kogelman et al. 2014).

Among clustering methods, one potent unsupervised technique is spectral clustering (Ezugwu et al. 2022; Xu and Tian 2015). This graph-based method considers the intrinsic geometric structure of data to overcome limitations in traditional clustering algorithms like K-means and hierarchical clustering (Von Luxburg 2007). It constructs a similarity graph from the data, which could be derived from various similarity measures, then performs dimensionality reduction by computing eigenvectors of the graph Laplacian matrix. These eigenvectors capture the data’s underlying structure, and subsets of the set of eigenvectors serve as lower-dimensional representations. By applying standard clustering algorithms like K-means to these representations, spectral clustering identifies clusters even in complex data with non-convex shapes and disconnected components, making it valuable for tasks like biological network analysis. However, despite the unsupervised characteristics of spectral clustering, users must pick how many clusters are expected from the data. We refer to such a choice as a *clustering regime*, and different choices lead to different groupings of the data, so that some data that share a cluster in one clustering regime are in different clusters in another. Although spectral clustering provides a metric on the importance of a clustering regime based on measuring gaps between consecutive eigenvalues (eigengaps), deciding which regime is best is subjective and up to the user. To overcome this, we introduce the *Multi-Layer Bundling (MLB)* method by combining the most prominent clustering regimes (based on eigengaps), each of which provides a particular viewpoint, to provide a comprehensive overview of the data clustering. The key idea is to use the intersection of clusters from several clustering regimes to form “bundles”. The number of clustering regimes used, starting from the most prominent, determines the layer number. Bundles of layer 1 are the clusters in clustering regime 1 (most prominent). A layer 2 bundle consists of points that were in the same cluster in clustering regimes 1 and 2. A layer 3 bundle consists of points that were in the same cluster in clustering regimes 1, 2, and 3. Since the bundling algorithm is based on recursive intersections, the number of elements in the bundles decreases as the layer number increases.

This technique not only reveals more refined clustering outcomes than a single spectral clustering regime but can also be particularly advantageous for understanding biological networks’ hierarchical organization, thereby providing a global to local view. It can also identify bridge elements mediating communication between network components.

To illustrate the procedure, we construct branched networks with varied amounts of branching. We then synthesize sample-feature data at each node based on the connectivity of the network. We determine the multiple layers of bundles of features from the correlation matrix of features based on the synthetic data. Finally, by projecting the results on the simulated network, we show the advantages of MLB over simple spectral clustering.

One advantage of the bundling method is that it is easy to perform, and does not rely on user-defined parameters. It also does not modify the data to facilitate analysis. The different bundling layers only reflect the combination of different spectral clustering regimes. These different viewpoints can help uncover relationships in the data that may not be apparent in a single application of spectral clustering.

By utilizing MLB, critical bridge sets of points of an interacting system can be uncovered. These bridge sets are crucial components that, if removed, would disconnect system compartments and disrupt information propagation. The tool’s capability to identify potential bridges deeply hidden in the data adds to its effectiveness.

Not only does the bundling method determine bundles of related elements, its iterative nature also provides the ability to form networks of bundles that indicate the inter-relationship of the different bundles. This information, contained in the *bundle co-cluster matrix*, is obtained through the layering process, and therefore not available in a standard spectral clustering process or other clustering methods (including tools in gene co-expression analysis) (Von Luxburg 2007; Ezugwu et al. 2022; Xu and Tian 2015; Van Dam et al. 2017).

In summary, the MLB method presents several advantages over standard spectral clustering for deciphering relationships among elements of biological networks and other complex datasets. It is built on spectral clustering and is readily implemented. We demonstrate the technique here using synthetic data in which the network of correlations is prescribed. It is intended to be used, however, for clustering any biological data in which a similarity matrix can be constructed.

## Methods

### Network Construction and Data Synthesis

Correlated biological data often reflect an underlying structural network. For example, metabolites are related through metabolic enzymes, and genes through transcription factors. To test the bundling method, we start with a structural network and use it to generate synthetic data. The advantage to doing this is that we know, and can manipulate, the structural network that gave rise to the data, so we can measure how well the method captures properties of that network.

**Fig. 1:**
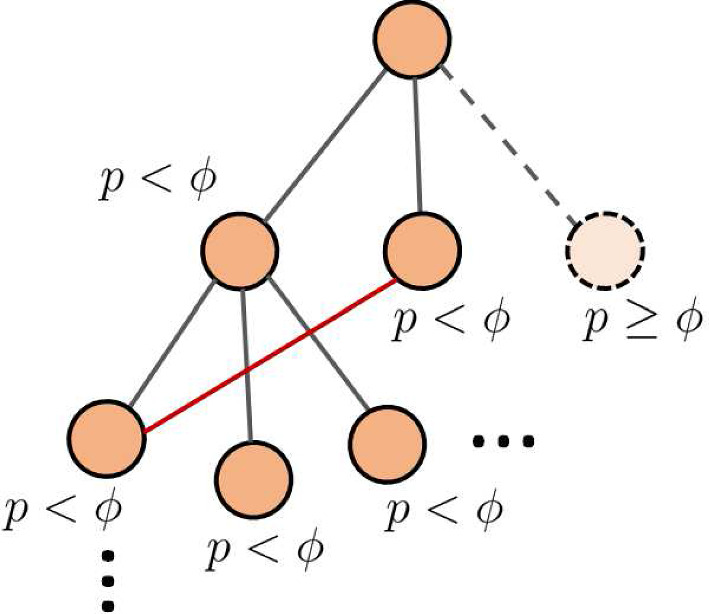
Construction of a structural network is based on branching parameter $\phi \in [0,1]$. In this process, child nodes are added to a parent if a pseudo-random number (*p*) is less than $\phi$. This process generates a rooted tree, and is stopped when there are 500 nodes. At this point, random edges are added to the network to create cycles (red line) (Color figure online)

The network construction begins with a single node. A new node is added and connected to the first node with probability $\phi$, where $\phi$ is referred to as the branching parameter. A pseudo-random number is drawn from a uniform distribution over the interval [0, 1]. The new node is added if the random number is less than $\phi$. The neighbor addition continues until the next drawn random number is $\ge \phi$. This process is applied to each node as it is added to the network (Fig. 1) until there are $n=500$ nodes.

**Fig. 2:**
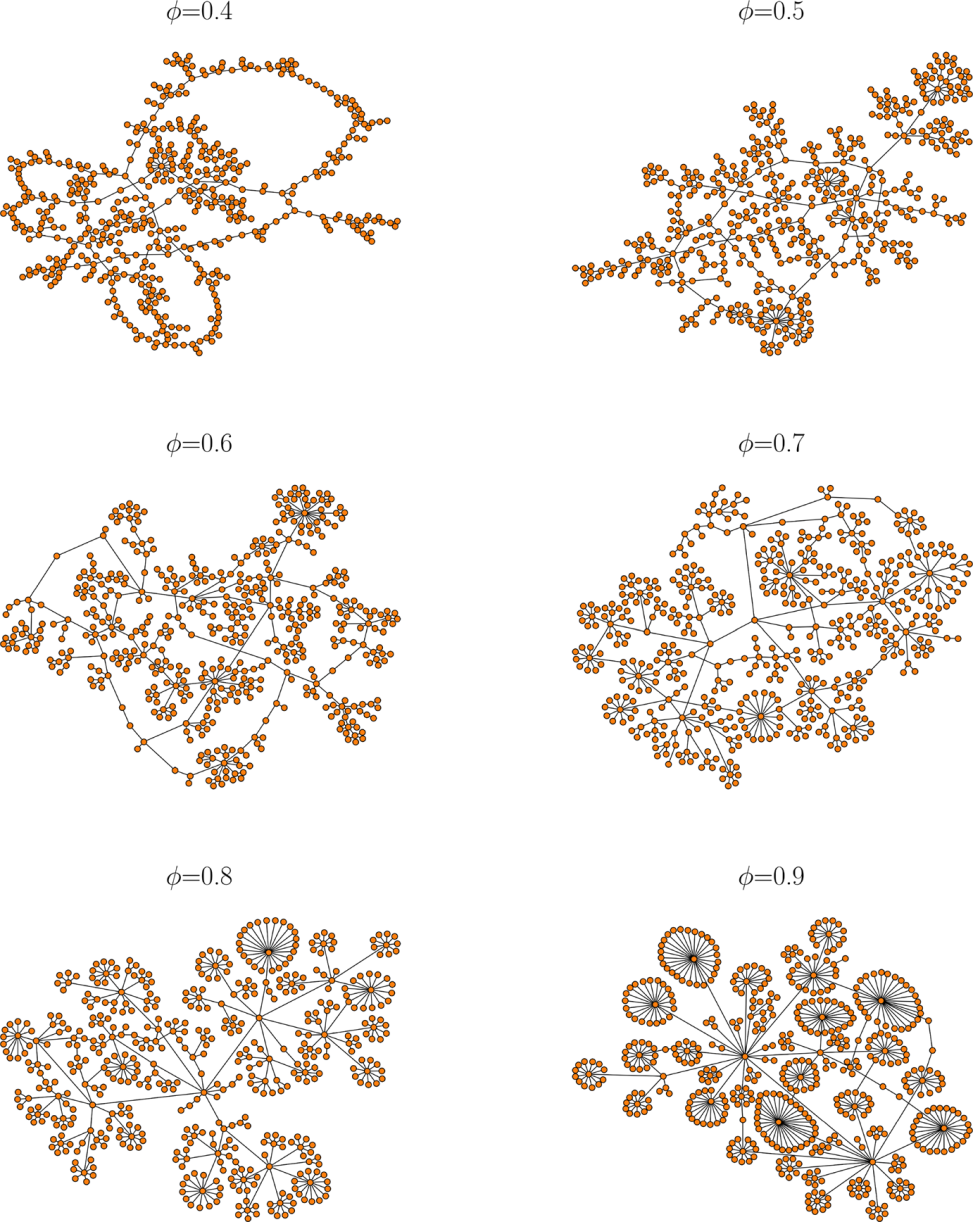
Examples of structural networks for different branching parameter values (Color figure online)

After establishing the initial network, a rooted tree with given probability of branching, we randomly add ten additional edges to form cycles. Figure 2 shows examples of six networks constructed this way with different values of the branching parameter. When the branching parameter is low, nodes have similar degrees. When high, there is a wide distribution of degrees, with some nodes serving as network hubs. Half of the edges in the network, chosen at random, are labeled as “$+$” and the other half as “−”.

The second phase of the synthetic data generation involves assigning values to the nodes in the network. To start, a node is chosen at random and given a value, which can be positive or negative. As long as no neighboring nodes have assigned values, their value is drawn from a normal distribution ${\mathcal {N}}(\mu =0, \sigma )$ for a given $\sigma$. If neighboring nodes have assigned values, the current node’s value is based on the values of neighboring nodes and the sign of the labels on the connecting edges.

**Fig. 3:**
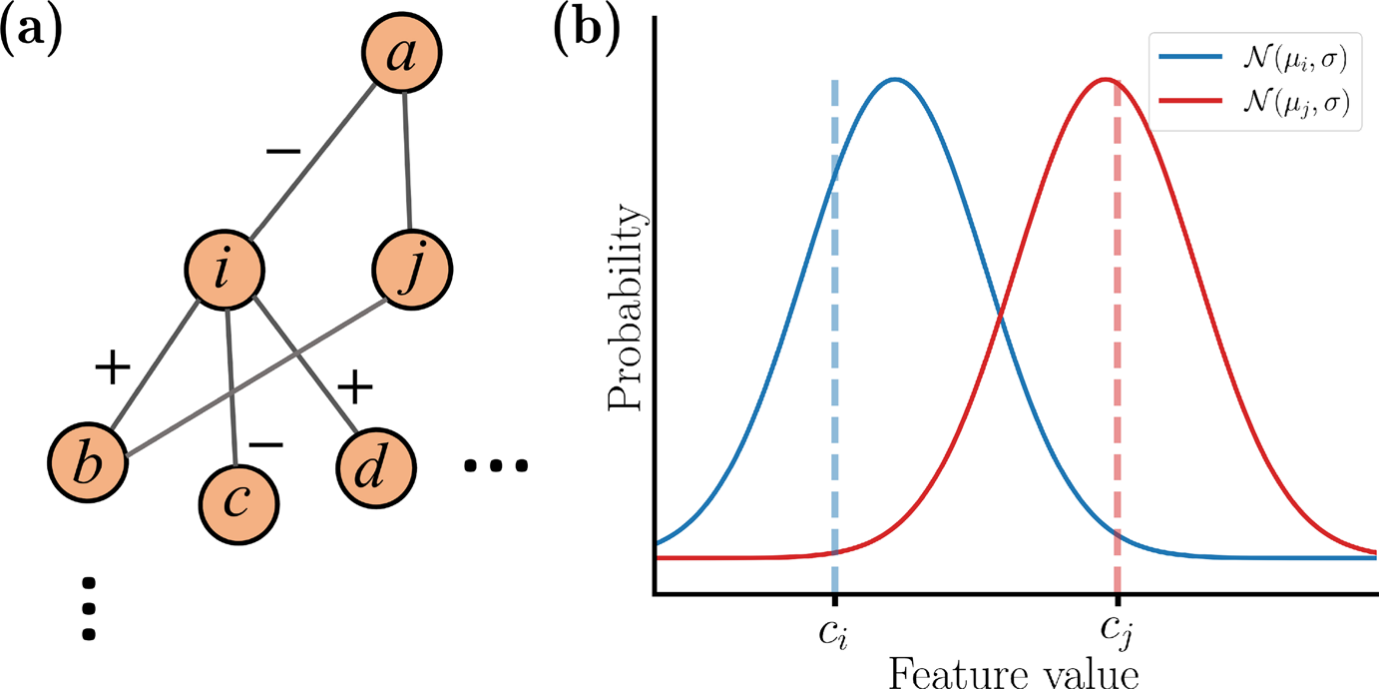
Illustration of the elements that go into the assignment of feature value for node *i* (Color figure online)

An example of the process is shown in Fig. 3. Suppose that values for nodes *a*, *b*, and *c* have been assigned, and that a value for *i* is next to be determined. The initial step in the determination of the value $c_i$ is to choose a value $\mu _i$ that is based on the values assigned to neighbors and the edge polarities. In this example, $\mu _i =(c_b - c_a - c_c)/3$. (The feature value from node *d* is not included since it has not yet been assigned.) The feature value for node *i* is then determined by drawing a real number from the normal distribution ${\mathcal {N}}(\mu _i, \sigma )$. In general, for any node *i* the formula for $\mu _i$ is:

1 $$\begin{aligned} \mu _i=\sum _{j\in N^*_i}\dfrac{\epsilon _j c_j}{|N^*_i|} \end{aligned}$$

where $|N^*_i|$ is the cardinality of the set of assigned neighbors of node *i* and $\epsilon _j$ reflects the edge polarity ($\epsilon _j = 1$ if polarity is positive, and $\epsilon _j = -1$ otherwise). We maintain a fixed $\sigma$ for the normal distribution throughout this process. However, the performance of the bundling method is tested for different $\sigma$ values.

Finally, for a given network of *n* nodes, we form the feature matrix, $\textbf{S}_{m\times n}$, by iterating the value assignment process a total of $m=400$ times. This method for producing synthetic data provides a dataset with a large variation of values for each node while maintaining the prescribed positive or negative correlations among neighboring nodes.

### Overview of Spectral Clustering

We begin with an overview of spectral clustering (Von Luxburg 2007) since this is the foundation of our bundling approach. Since we are using a correlation-based metric, we begin with the Pearson correlation matrix $\textbf{R}$ from the sample-feature matrix $\textbf{S}$. This can be used to construct a weighted network with edge weight equal to the absolute value of the correlations, described by the affinity matrix $\textbf{A}=|\textbf{R}|$. Spectral clustering uses the graph Laplacian ($\textbf{Q}$) of this matrix:

2 $$\begin{aligned} \textbf{Q}:=\textbf{D}-\textbf{A} \end{aligned}$$

where $\textbf{D}$ is the diagonal “degree matrix”

3 $$\begin{aligned} \textbf{D}_{i,i}=\sum _{j\ne i} \textbf{A}_{i,j}. \end{aligned}$$

To remove the dependency on data size, we normalize the Laplacian matrix:

4 $$\begin{aligned} \widehat{\textbf{Q}}:=\textbf{D}^{-1/2}{} \textbf{Q} \textbf{D}^{-1/2} \end{aligned}$$

$\widehat{\textbf{Q}}$ is positive semi-definite and has *n* non-negative real-valued eigenvalues that can be labeled in ascending order $0=\lambda _1 \le \lambda _2 \le \cdots \le \lambda _n$ and corresponding eigenvectors $\textbf{v}_1, \textbf{v}_2, \ldots , \textbf{v}_n$. A key step in spectral clustering is identifying gaps in the size of eigenvalues. We define $\delta _i$ as the difference between consecutive eigenvalues, or eigengaps:

5 $$\begin{aligned} \delta _i:= \lambda _{i+1}-\lambda _{i}, \;\; i=1, 2, \ldots , n-1. \end{aligned}$$

These can then be ordered,

6 $$\begin{aligned} \delta _{\kappa _1}\ge \delta _{\kappa _2}\ge \cdots \ge \delta _{\kappa _{n-1}}. \end{aligned}$$

where the set of $\kappa$ values are eigenvalue indices:

7 $$\begin{aligned} \{\kappa _1, \kappa _2, \ldots , \kappa _{n-1}\}. \end{aligned}$$

A large eigengap indicates a natural break in the data, and the associated index indicates the number of clusters. The most prominent break in the data occurs with $\kappa _1$ clusters, the second most prominent with $\kappa _2$ clusters, etc. The eigenvectors $\textbf{v}_1,\ldots , \textbf{v}_{\kappa _i}$ span a subspace of ${\mathbb {R}}^n$ and each *n*-dimensional data vector is projected into this subspace at this *i*th “clustering regime”. Now, we need a label assignment algorithm to cluster the projected data points into the clustering regimes *i*. To do this, we use the “cluster-qr” algorithm (Damle et al. 2018) implemented in the scikit-learn Python package (Pedregosa et al. 2011). The outcome clustering regime *i* is represented as a set of sets:

8 $$\begin{aligned} C_{i}:=\{\xi ^i_1,\ldots , \xi ^i_{\kappa _i}\} \end{aligned}$$

where $\xi ^i_j$ is the set consisting of the *j*th cluster in regime *i*. We then define a “layer $\ell$ bundle $\beta$” as an intersection of $\ell$ clusters $\xi _j$s, each belonging to a clustering regime from layer 1 to layer $\ell$. That is, a bundle of layer $\ell$ is the set of points that were in the same cluster for each clustering regime $1,...,\ell$. The set of bundles of layer $B_\ell$ is then

9 $$\begin{aligned} B_\ell :=\{ \beta \;|\;\beta =\bigcap _{i=1}^{\ell }\xi ^i_{j_i}\ne \varnothing ,\;j_i=1,\ldots ,\kappa _i\}. \end{aligned}$$

## Results

### Visualization of Clustering Regimes and Bundles Within the Network

**Fig. 4:**
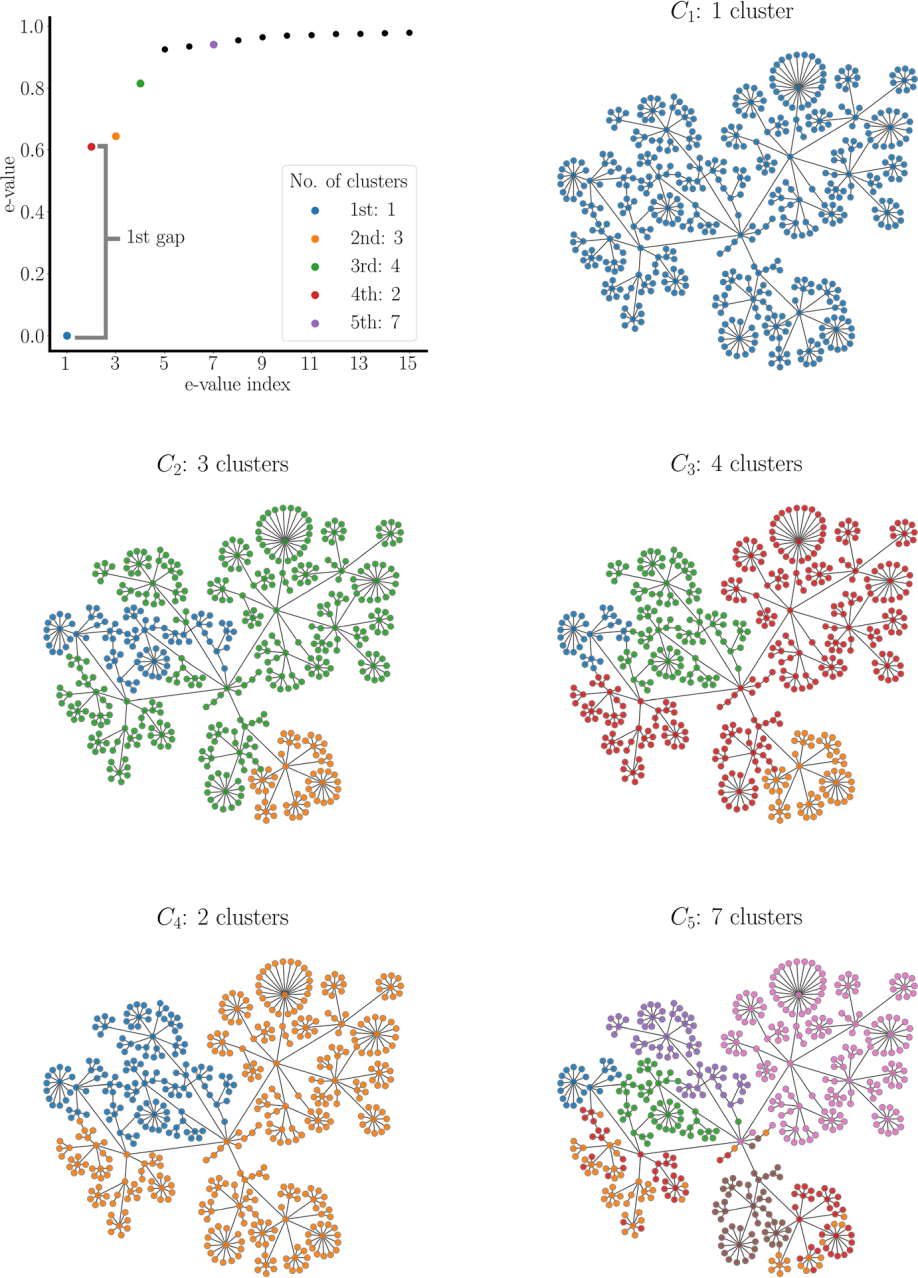
Five clustering regimes from synthetic data generated as described in Methods, with parameters $\phi =0.8$ and $\sigma =0.2$. Eigengaps in the sorted eigenvalues of the graph Laplacian provide the five best options for the number of clusters in different clustering regimes. In each clustering regime, the nodes are colored based on the clusters that they fall into (Color figure online)

Figure 4 shows a structural network used to compute synthetic data for a feature matrix $\textbf{S}_{m \times n}$, as described in Methods. The nodes are color-coded according to the spectral clustering of these data. The plot shows the graph Laplacian eigenvalues sorted in ascending order from smallest (zero) to largest, with only the first 15 displayed. The first eigengap is between the first eigenvalue and the second, which suggests that the most prominent cluster is the entire network, so there is a single cluster in clustering regime 1 ($C_1$). The next largest gap is between eigenvalues $\lambda _3$ and $\lambda _4$, so this clustering regime ($C_3$) has 3 clusters. A few additional clustering regimes are also shown in the figure, each clustering the network in different ways. No pattern in the clusters appears among cluster regimes $C_2$ to $C_5$. In particular, the number of clusters does not necessarily increase or decrease monotonically as one moves through the clustering regimes. Even though certain clustering regimes bear similarities or subset relations, this is not necessarily the case. Each clustering regime is a different projection of the data into spaces spanned by a different number of eigenvectors, so data points that are clustered together in one projection may be split apart in a different projection.

**Fig. 5:**
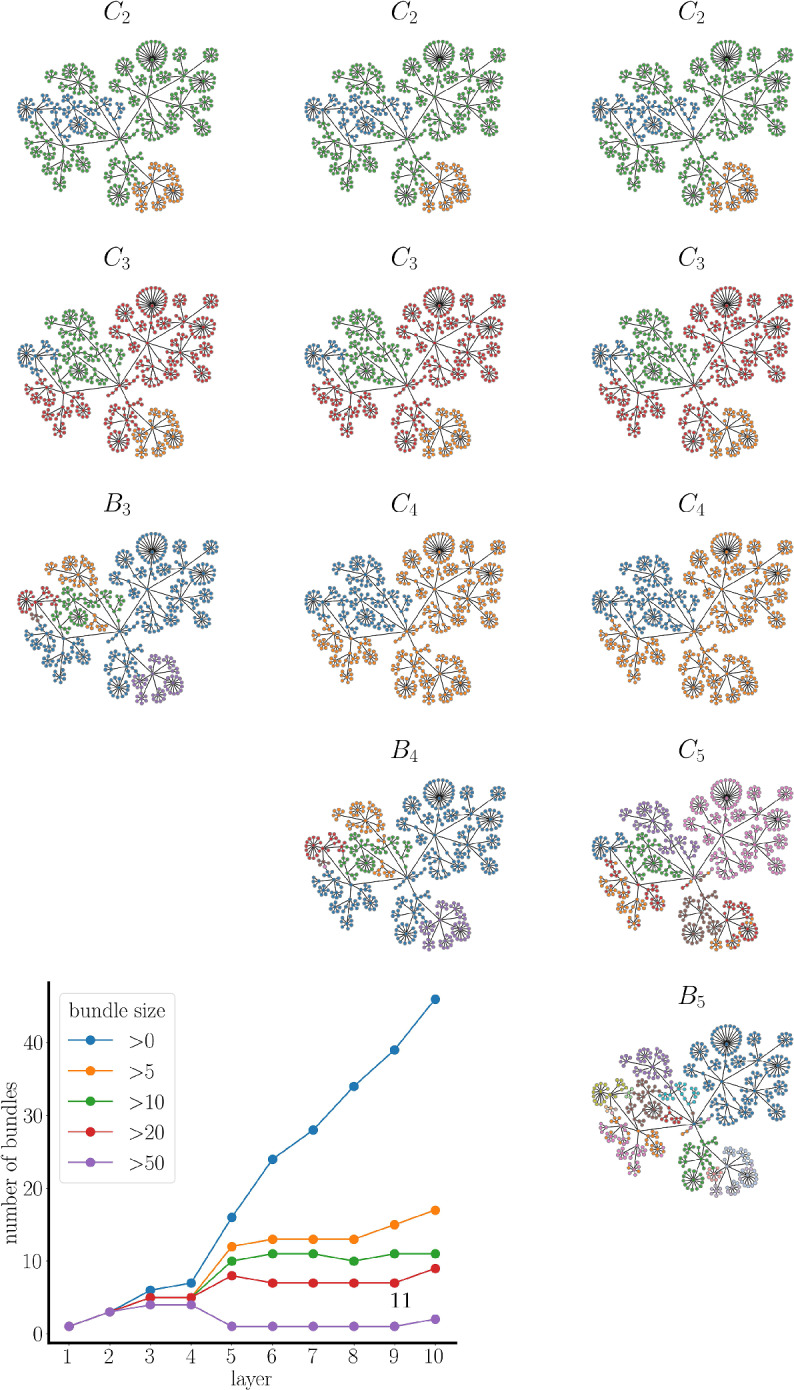
Three bundling layers and the clustering regimes from which they are formed, arranged as columns. Nodes of the same color are in the same cluster of a clustering regime ($C_j$) or the same bundle of a bundle layer ($B_k$). The plot shows the number of bundles of various sizes versus the layer number. The blue curve is the total number of bundles at each layer (Color figure online)

Those nodes that stay together across clustering regimes have a high degree of similarity, and we say that they are elements of the same bundle. Figure 5 depicts three layers of bundling from the same set of data used in the previous figure. The set of bundles of layer 3, $B_3$, consists of 5 bundles (color coded, left column). All nodes in one bundle were in the same cluster in $C_2$ and in $C_3$ (the number of bundles will never be less than the largest number of clusters in the composite clustering regimes). The set of layer 4 bundles, $B_4$, consists of 6 bundles (center column), which is one more than in layer 3. This increase occurs because another clustering regime, $C_4$, is used in addition to the two regimes used before. The number of bundles increases further, to 16, in layer 5 (right column). Adding additional clustering regimes can never decrease, and will likely increase, the number of bundles. By incorporating more clustering regimes, i.e., delving into deeper layers of bundling, we obtain more refined bundles, offering a more localized view of the data. Therefore, MLB provides a systematic transition from a global view (the sole layer 1 bundle contains all data points) to a local view of the data.

How many layers of bundling should one form? The deepest possible layering is equal to the number of nodes. At this level, all bundles contain just a single node, so no useful information is provided. As depicted in the plot in Fig. 5, we can identify suitable points where stopping would be prudent. The figure shows the cardinality of bundles of different sizes versus layer number. The blue line is the total number of bundles regardless of size. This is a non-decreasing function of the bundling layer, as mentioned above. The orange line shows the number of bundles with at least 5 elements. The purple line shows the number of bundles that are the largest, with over 50 elements each. The total number of bundles increases consistently from layer 4 onward, as the larger bundles from prior layers break into smaller ones (the purple curve of large bundles drops to 1 after layer 4). However, the bundle size distribution remains relatively constant from layer 5 until layer 8 as the orange, green, red, and purple curves reach a plateau. This suggests that layer 5 would be a good stopping point.

### Multi-layer Bundling Depicts Bridge Sets of Data

One use of MLB is the identification of *bridge sets* within a given dataset, which are nodes that connect one large module of data to another large module. A bridge set can be viewed as transitional data which shares information between the two larger modules, and would typically be a smaller set of data than those that it couples together. In the structural network used to generate the synthetic data, such a bridge should be a small set of nodes that connects to much larger sets of clusters or bundles. To quantify this, we define a *bridge factor* of a cluster or a bundle *x* by the ratio of the cardinality of each neighboring cluster/bundle to that of *x*, $N_x$. That is:

10 $$\begin{aligned} \gamma _x= {\left\{ \begin{array}{ll} \prod _{y \in N_x}\dfrac{|y|}{|x|},&{} \text {if } |N_x| > 1\\ 0,&{} \text {otherwise }. \end{array}\right. } \end{aligned}$$

where *x* is a bundle or a cluster, $|\cdot |$ is cardinality, and $N_x$ is the set of neighboring bundles or clusters. If a cluster/bundle has only one neighbor ($|N_x|=1$), then its bridge factor is zero.

**Fig. 6:**
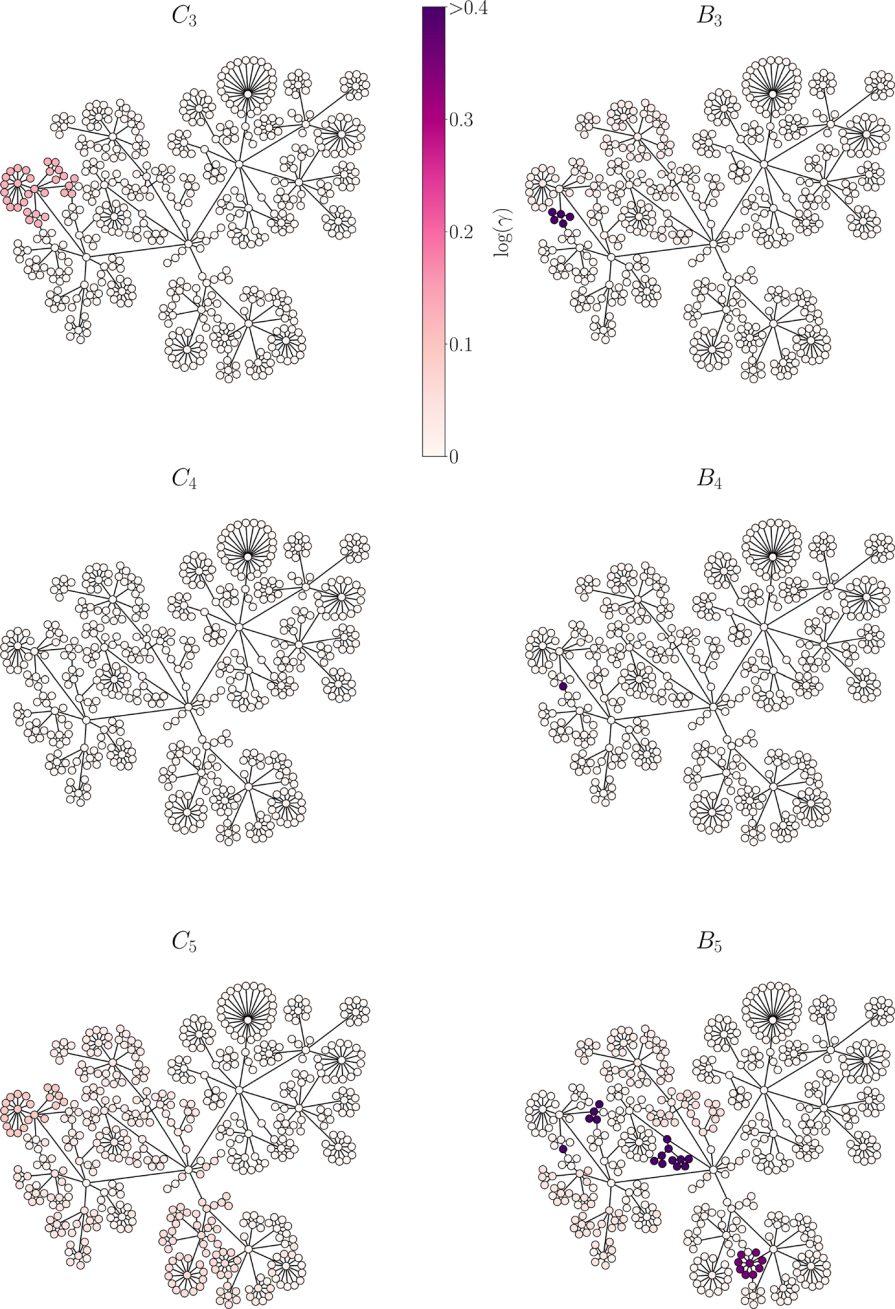
Bundling more effectively extracts bridge sets of data than clustering. In the left column, nodes are colored corresponding to the bridge factor value of clustering regimes $C_3$, $C_4$, and $C_5$. The same is shown on the right but for bundling layers $B_3$, $B_4$ and $B_5$. Darker colors mean larger $\gamma$, and are elements of the bridge sets (Color figure online)

Figure 6 shows a comparison between the bridge factors of layers $B_3$, $B_4$ and $B_5$ with their corresponding clustering regimes $C_3$, $C_4$ and $C_5$. The dark color indicates high values of $\gamma$, and the light color indicates low values. The figure shows that some bundles have high bridge factors (dark colored bundles in right column). These bridges are small groups of nodes that are identified as bridges because they clustered together with neighboring nodes in some clustering regimes, but not all, and the cardinality of the bridge set is small relative to that of its neighbors. In contrast, the bridge factors for clusters (left column) are much lower (lighter color), and therefore the bridge sets of data are not as clearly defined.

**Fig. 7:**
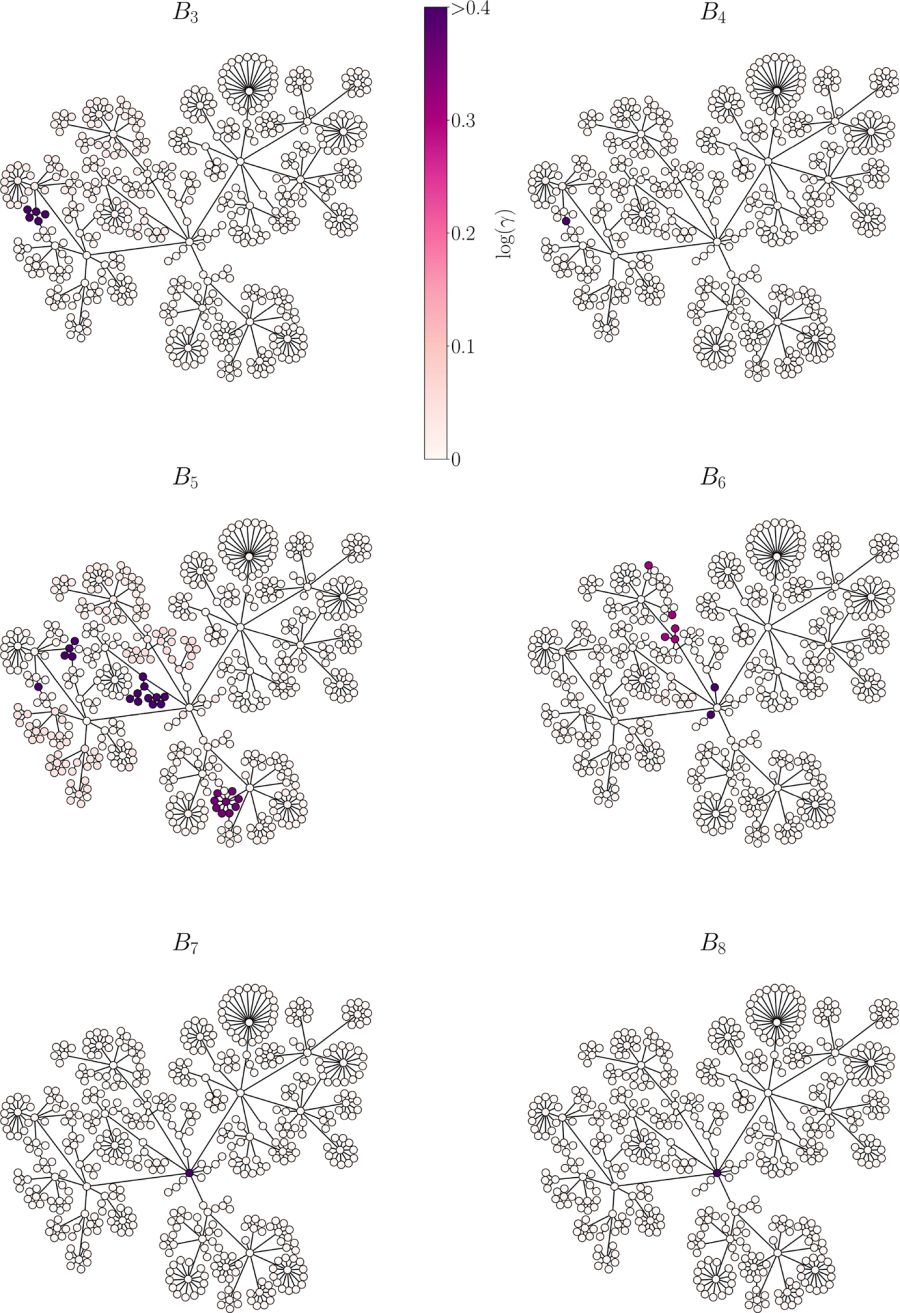
Local bridge sets are identified at low bundling layers, while global bridge sets are identified at higher layers. The nodes in the six bundling layers are colored according to the value of the bridge factor $\gamma$, with dark color indicating large $\gamma$ (Color figure online)

A local bridge set between two other sets of nodes in a network can be thought of as providing a short path between these other sets, although another path may exist. A global bridge set provides the only path between sets of neighboring nodes. At low layers of bundling, local bridge sets are identified (see $B_3$–$B_5$ in Fig. 7), while global bridge sets are identified in later layers (see $B_6$–$B_8$ in Fig. 7). In both layers $B_7$ and $B_8$, the only bridge set identified is the single node in the center of the network that connects all modules (it has the highest betweenness centrality). The ability to identify bridge sets at different scales reflects the multi-scale nature of the bundling method.

**Fig. 8:**
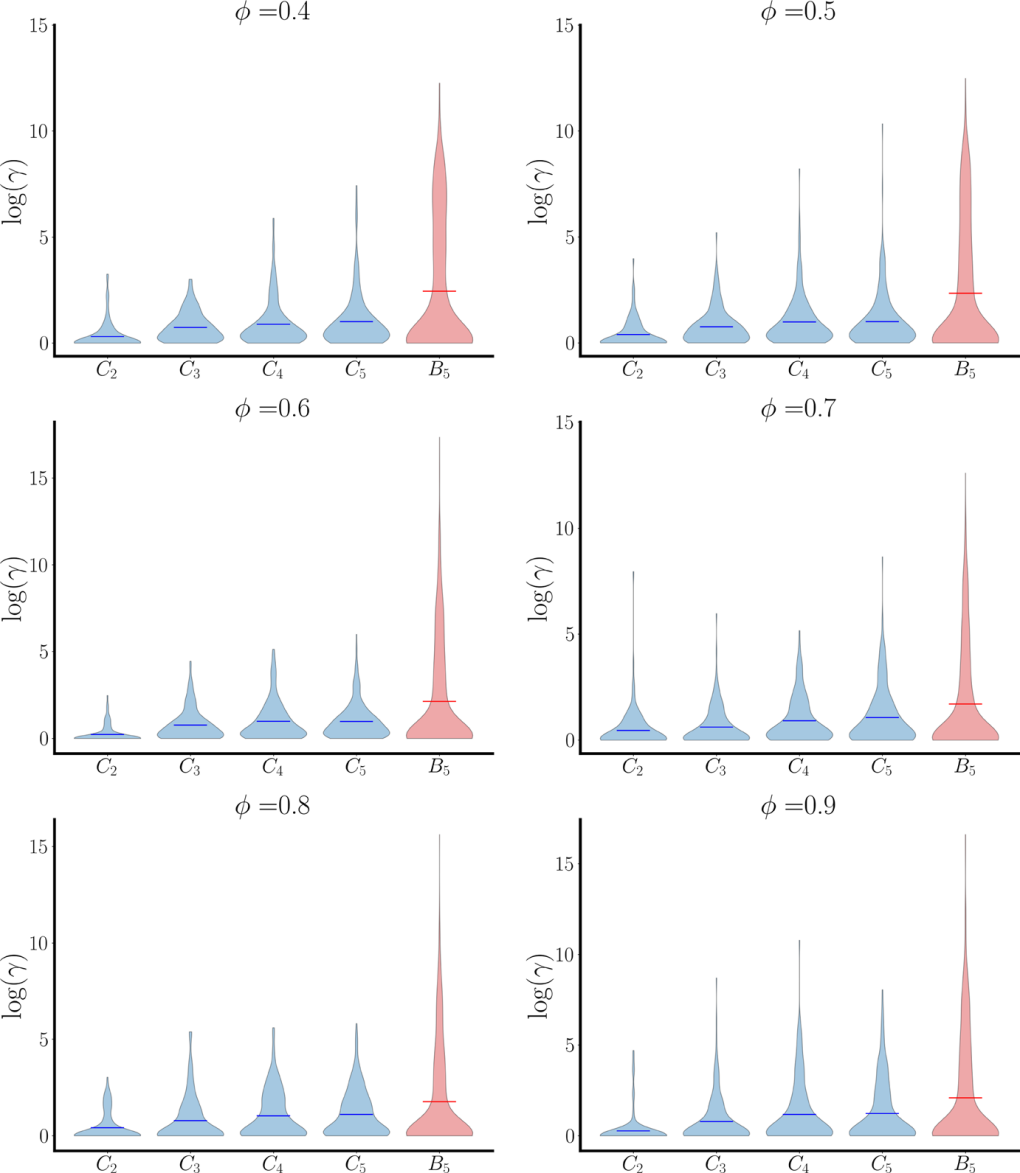
Violin plots of bridge factors computed from synthetic data generated by 50 structural networks with 6 different branching parameters $\phi$. Bundle layer 5 is shown, along with its composite clustering regimes. Bridge factors in the upper tails reflect bridge features (Color figure online)

The previous examples suggest that bridge sets of data are better detected in bundles than clusters. To determine if this is true in general, we examined synthetic data generated by 50 different structural networks with branching parameters $\phi =0.4,0.5,\ldots ,0.9$. In Fig. 8, for each branching parameter, the violin plots of bridge index values for clustering regimes 1 to 5 and bundle layer 5 ($B_5$) are plotted. Any distinct bridge has a very high $\gamma$, putting it in the upper tail of the violin plot. For all values of the branching parameter, the tail is longer and thicker for $B_5$ than for the clustering regimes, indicating that there are more large bridge factors in bundle layer 5 than in the clustering regimes from which it is computed, confirming that bridge sets of data are better identified by bundling than by spectral clustering alone.

### The Network of Bundles Can Help Determine the Structure of the Feature Correlation Network

**Fig. 9:**
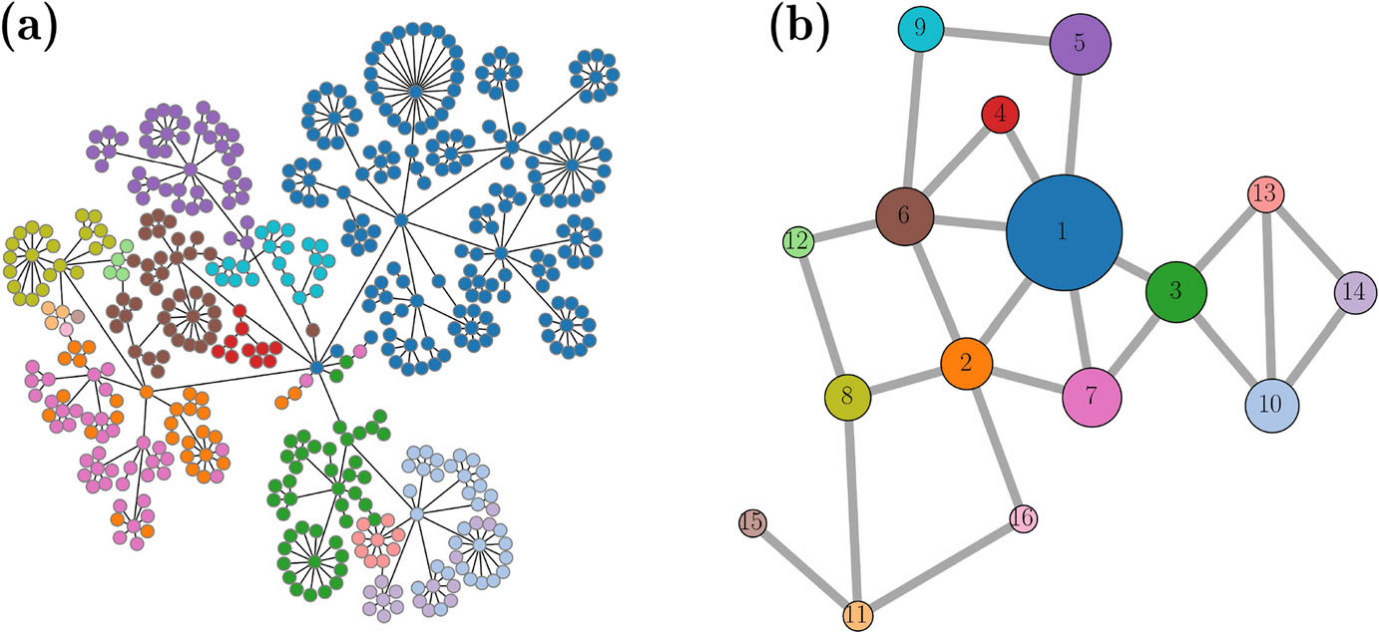
An example of a structural network used to generate synthetic data and corresponding layer 5 bundles. **a** The structural network with nodes colored according to which layer 5 bundle they are in. **b** The corresponding bundle network, where an edge means that at least one node of one bundle is connected to a node in a neighboring bundle in the structural network on the left (Color figure online)

One property of the bundling process is that it is iterative, with each iteration building on the last and incorporating the next most prominent spectral clustering regime. If two nodes are in different bundles in layer 5, they could have been in the same bundle in layer 4. In fact, this happens frequently with some bundles: they are closely related to one or more other bundles in that they have many nodes that were together in an earlier bundling layer. This bundle overlap can be used to construct a *bundle network*, whose nodes are bundles and the edges indicate significant bundle overlap. We show next that this bundle network can help determine similarities in the data. That is, it can help find the network of interactions (e.g., the structural networks in Fig. 2) that led to the correlations among the data. As an example, consider the network shown in Fig. 9a. The nodes in this network are colored according to the layer 5 bundles that they are part of. It is clear from this that the nodes of each bundle are co-localized in the network. This is expected since the data values were chosen to correlate (or anti-correlate) with neighboring nodes. The graph in Fig. 9b is the bundle network that corresponds to the structural network on the left. Two bundles are connected by an edge if one node in one bundle is connected to a node in the other in the structural network on the left. The challenge is to find the edges in the bundle network without knowing the structure of the network used to generate the data on the left. Our goal is to use the information obtained from spectral clustering and bundling to find these edges, and thus understand how the different bundles are interconnected. We first attempt to reconstruct the bundle network using only bundle affinity properties derived from the affinity matrix of the data, $\textbf{A}$. Then, we use the bundle overlap (co-clustering) information obtained through the layering process. Finally, we examine their combined effect on network reconstruction.

**Fig. 10:**
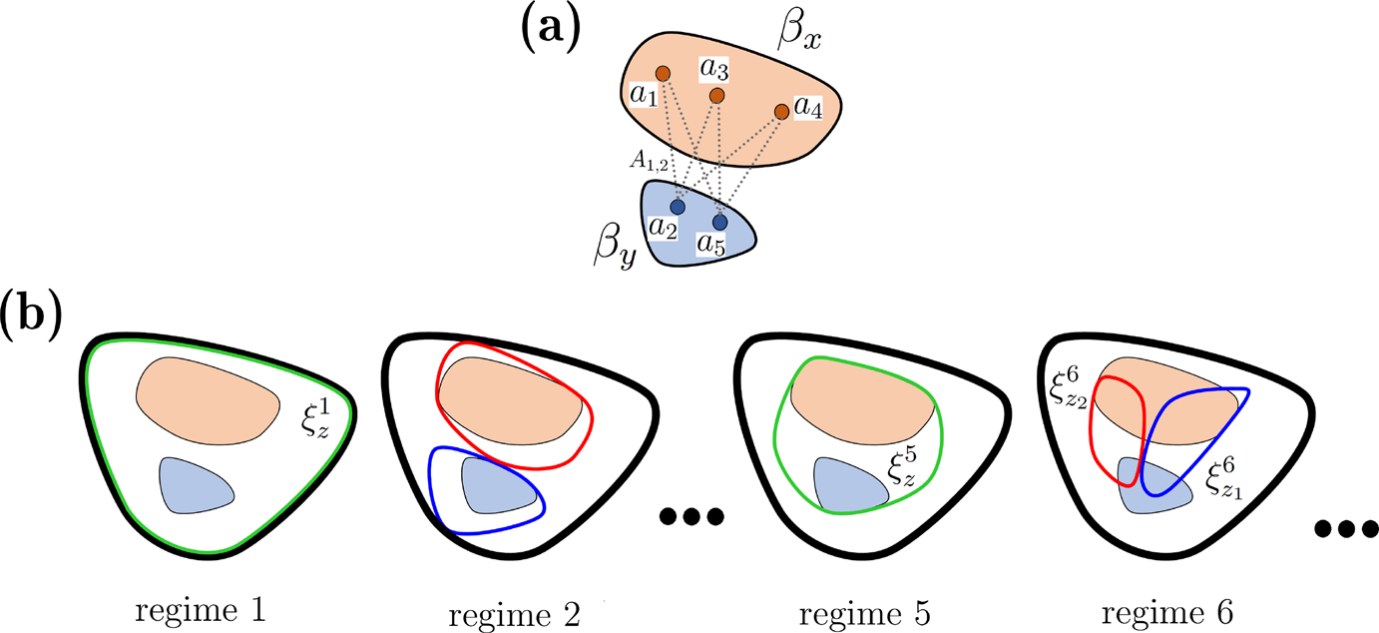
**a** Illustration of how element (*x*, *y*) of the bundle affinity matrix $\textbf{H}^k$ is computed. The orange and blue sets indicate two bundles, $\beta _x$ and $\beta _y$. Each dashed line segment indicates an element of the affinity matrix that is used in the calculation. In this example, the six affinity values are averaged to give $\textbf{H}^k_{x,y}$. **b** Illustration of how element (*x*, *y*) of the bundle co-cluster matrix $\textbf{L}^5$ is computed, using the same two bundles. The black closed curve indicates the entire data set (nodes in the structural network), which is the regime 1 cluster that contains elements of $\beta _x$ and $\beta _y$. The closed red, blue, and green curves indicate clusters at different layers. A cluster at regime *k* that contains elements of both $\beta _x$ and $\beta _y$ is denoted by $\xi ^k_z$ (Color figure online)

We begin by constructing a square symmetric matrix $\textbf{H}^k$ corresponding to bundle layer *k* whose dimension is equal to the number of bundles at this layer, which we denote as $N^k$. Element (*x*, *y*) in $\textbf{H}^k$ is the average of the affinity values between nodes in these bundles. This is illustrated in the top diagram in Fig. 10, where a line segment connecting nodes *i* and *j* corresponds to the *i*, *j* element of the affinity matrix $\textbf{A}$ (whose elements are the absolute values of the Pearson correlation between feature values, as defined in Methods). The 6 segments correspond to the 6 elements that are averaged to calculate $\textbf{H}^k_{x,y}$. The general formula for element (*x*, *y*) of this *bundle affinity matrix of layer k* is:

11 $$\begin{aligned} \textbf{H}^{k}_{x,y}=\dfrac{\sum _{i\in \beta _x,j\in \beta _y}{} \textbf{A}_{i,j}}{|\beta _x||\beta _y|} \end{aligned}$$

The algorithm to construct what we call the “H-bundle network” has two steps. In the first step, the maximum value along each row of $\textbf{H}^k$ is determined (in case of a tie, we use the location of the maximum with the smallest index number). The indices for each of these maxima are used to connect two bundles by an edge. Since there are $N^k$ rows in the matrix, there will be at most $N^k-1$ edges. The resulting H-bundle network may have more than one connected component. If this is the case, then a second step is taken to add additional edges. In this step, all elements of $\textbf{H}^k$ not already used for edges are listed in descending order according to their magnitude. If the indices of the top element would provide an edge that reduces the number of connected components in the network then this edge is added to the network. If not, it is discarded and the next element in the list is examined. This process continues until there is a single connected component in the H-bundle network.

**Fig. 11:**
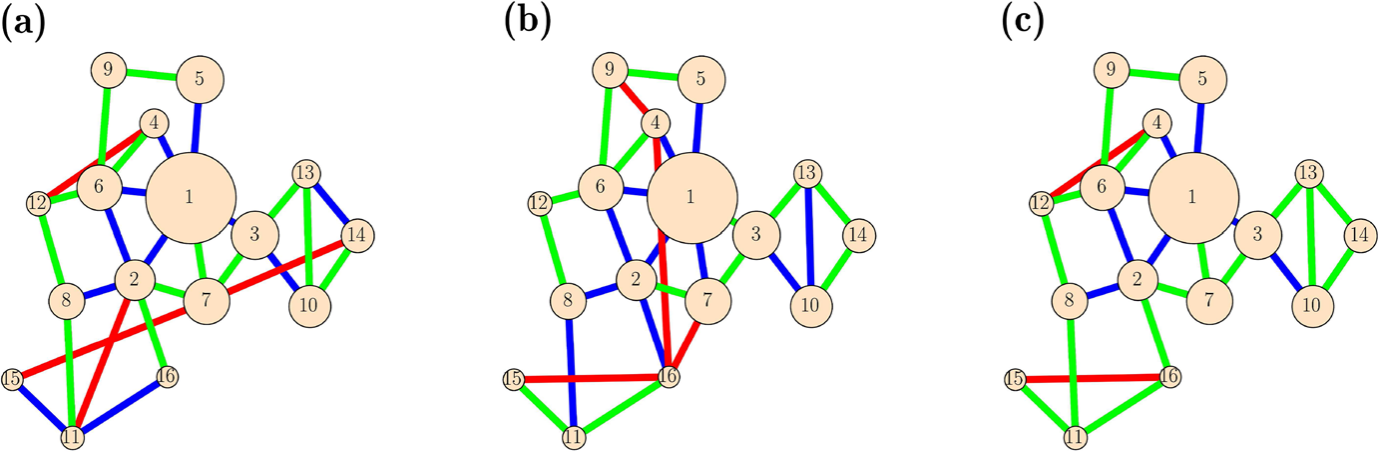
Three approaches for reconstituting the bundle network of Fig. 9b using only spectral clustering and bundling information. **a** The H-bundle network constructed using the bundling affinity matrix of layer 5, $\textbf{H}^5$. **b** The L-bundle network constructed using the layer-5 bundle co-clustering matrix $\textbf{L}^5$ with $r=12$. **c** The LH-bundle network constructed using the matrix $\mathbf{L+H}$ with $r=12$. In all networks, the green edges match edges in the target bundling network of Fig. 9b. Blue edges are in the target network, but not the reconstituted network (i.e., false negatives). Red edges are in the reconstituted network, but not the target network (i.e., false positives) (Color figure online)

The H-bundle network constructed this way is shown in Fig. 11a. The green edges are those that match the target bundle network of Fig. 9b. Many edges in the target were missed in the construction of the H-bundle network, and these missing edges (false negatives) are indicated in blue in Fig. 11a. Finally there are some edges in the H-bundle network that are not in the target network. These false positives are shown in red in Fig. 11a.

Another option for determining the inter-relationship of bundles is to see how they cluster together across layers. This information is used to form a square symmetric matrix $\textbf{L}^k$ corresponding to bundle layer *k*, with dimension equal to the number of bundles at this layer, $N^k$. We call this the *layer-k bundle co-cluster matrix*.

The calculation of element (*x*, *y*) of the co-cluster matrix $\textbf{L}^5$ is illustrated in Fig. 10b. At each clustering regime less than or equal to 5, the cluster or clusters that each bundle belongs to are identified and their cardinality determined. For example, at clustering regime 1, there is a single cluster containing both bundles (it contains all data points), and this cluster is denoted $\xi ^1_z$ with cardinality $|\xi ^1_z|$ equal to the number of data points *n*. The sum of the cardinality of $\beta _x$ and $\beta _y$ is then divided by $|\xi ^1_z|$ to obtain the fraction of the cluster occupied by the two bundles. This is repeated at each regime up to and including clustering regime 5 and the values summed. At some regimes, the bundles will be in different clusters (as illustrated in Fig. 10b at regime 2), and in this case the fraction is set to 0. In general, for a calculation of bundles at layer *k*, this is

12 $$\begin{aligned} \sum _{\ell =1}^k\dfrac{|\beta _x|+|\beta _y|}{|\xi ^{\ell }_z|}, \end{aligned}$$

which will appear in the equation for $\textbf{L}^k_{x,y}$. The next step in calculating $\textbf{L}^k_{x,y}$ is to see how the bundle elements co-cluster as they break apart at higher clustering regimes. For the example shown in Fig. 10b, the two bundles $\beta _x$ and $\beta _y$ have elements in two clusters at regime 6, cluster $\xi ^6_{z1}$ and $\xi ^6_{z2}$. We compute the fraction of each of these clusters (and any others containing elements of both $\beta _x$ and $\beta _y$) occupied by the two bundles. This is repeated over a number of clustering regimes and the fractions summed together. Finally, this sum is added to the previous sum, so that element (*x*, *y*) of the bundle co-clustering matrix at layer *k* is:

13 $$\begin{aligned} \textbf{L}^k_{x,y}=\sum _{\ell =1}^k\dfrac{|\beta _x|+|\beta _y|}{|\xi ^{\ell }_z|}+\sum _{{\ell }=k+1}^r\sum _{\xi ^{\ell }_z\in C^{x,y}_{\ell }}\dfrac{|\beta _x \cap \xi ^{\ell }_z|+|\beta _y \cap \xi ^{\ell }_z|}{|\xi ^{\ell }_z|} \end{aligned}$$

where *r* is the total number of clustering regimes considered (we use $k=5$ and $r=12$ in the results shown below). We note that for cluster regimes ${\ell } \le k$ there is at most one cluster containing elements of both $\beta _x$ and $\beta _y$, but for regimes ${\ell }>k$ there may be several such clusters that form a set $C^{x,y}_{\ell }$. It is for this reason that the double summation is included in the second term of Eq. 13.

**Fig. 12:**
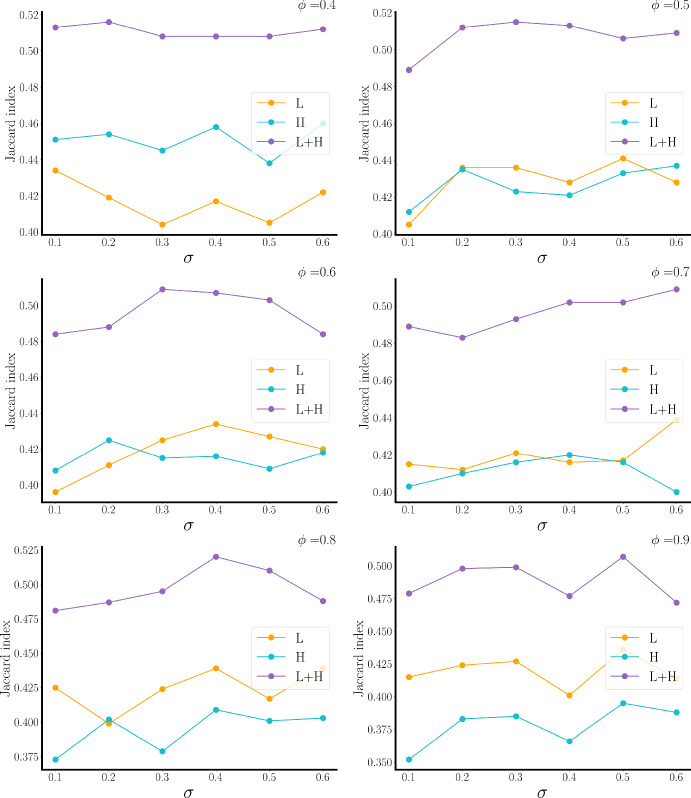
The performance of three different methods for reconstituting the target bundle network. The network branching parameter is $\phi$, and $\sigma$ is the standard deviation in the calculation of synthetic feature values. The orange curve indicates the match of the L-bundle network to the target network, the blue is the match of the H-bundle network, and the purple is the match of the LH-bundle network to the target (Color figure online)

Once the bundle co-cluster matrix of layer *k* is constructed, it is used to compute an “L-bundle network” using the same approach that was used for the H-bundle network. The L-bundle network for the example of Fig. 9 is shown in Fig. 11b. This L-bundle network matches 12 of the edges in the target bundle network of Fig. 9b, which is similar to the 13 correct edges in the H-bundle network. Once again, there are numerous false negative (blue) and false positive (red) edges.

While neither of these approaches outperforms the other, each finds correct edges that the other misses. What if the two approaches are combined? A simple way of doing this is to normalize the two matrices and then add them together. The procedure used to pick edges for the H-bundle and L-bundle networks is again used, forming the “LH-bundle network” (Fig. 11c). This combined network does a better job of matching the target bundle network of Fig. 9b, with 16 correct edges. There are, however, still 7 false negatives and 2 false positives, so the match to the target is not perfect. The quality of the match can be quantified with the Jaccard similarity index (Liben-Nowell and Kleinberg 2007), which provides a measure of the overlap between the edge sets of the two networks. This is defined as:

14 $$\begin{aligned} \text {Jaccard index}=\dfrac{|{\mathcal {E}}_r\cap {\mathcal {E}}_t|}{|{\mathcal {E}}_r\cup {\mathcal {E}}_t|} \end{aligned}$$

where ${\mathcal {E}}_t$ is the edge set for the target bundle network and ${\mathcal {E}}_r$ is that of the H-bundle, L-bundle, or LH-bundle network.

To determine the performance of the three methods for reconstituting the bundle network, we applied this reconstruction method to a large number of synthetic data sets calculated from different branching parameters $\phi$ and standard deviation values $\sigma$ for the calculation of data values. To be comparable, for each parameter set the layer-5 bundle network was constructed, as were the layer-5 H-bundle, L-bundle, and LH-bundle networks. The Jaccard similarity index was used to quantify the overlap between the target bundle network and the reconstituted network. The results of the analysis are shown in Fig. 12. For each parameter combination, the LH-bundle network showed greater similarity to the target bundle network than either the H-bundle or L-bundle network. The figure also shows that the H-bundle network is closer to the target than the L-bundle network when the branching is low, and the opposite is true when the branching is high.

### Comparison Between Multi-layer Bundling and clustering with WGCNA

Here, we compare the clustering results of the commonly used WGCNA (Weighted Gene Co-expression Network Analysis) technique (Langfelder and Horvath 2008) with those of MLB. The comparison is made first using synthetic data, and then using biological data.

Throughout this comparison, we exercised different parameter values in WGCNA while using the default functions. In the similarity matrix calculation, we used unsigned Pearson correlation on expression data and enforced a soft threshold by raising the similarity matrix elements to a power to achieve scale-free topology in the degree distribution of the similarity network. The result is then transformed into a Topological Overlap Matrix (TOM) to measure the network connectivity of a gene pair by considering not only their direct interaction but also their interactions with other genes. Finally, hierarchical clustering with the Dynamic Tree Cut method is used to detect clusters (called “modules” in the WGCNA notation).

Most of the procedures above depend on user input, including three key tuning parameters: (1) the exponent (PWR) to produce a scale-free topology, (2) the minimum module size (MMS) that sets a lower bound on the size of a module, and (3) the merging threshold (MRT $\in [0,1]$) for merging the preliminary modules into a smaller set of modules.

**Fig. 13:**
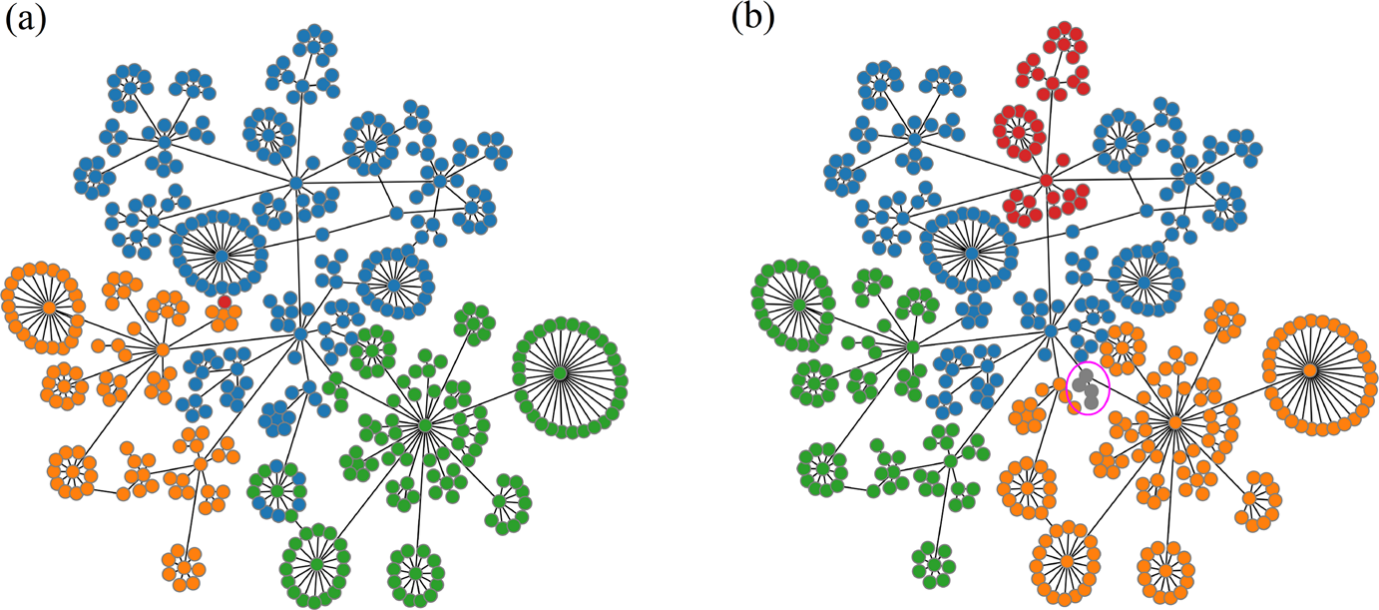
MLB versus the WGCNA clustering method. **a** Color-coded bundling of simulated test data from layer 3. **b** Color-coded modules of the same data using WGCNA with MMS = 20, PWR = 12, and MRT = 0.4 (Color figure online)

Using data from a synthetic correlation network, shown in Fig. 13, we do a comparison of the two methods. The left panel shows the bundling results for layer 3, while the right panel shows WGCNA clustering with the MRT parameter chosen to produce a number of modules that is similar to the number of bundles. There is a great deal of similarity between the clustering results, but also some differences. One difference is the large blue bundle is split into two modules (red and blue) in WGCNA. This splitting is not seen with bundling, even at different layers, and is peculiar since it splits the blue module into separate uncoupled components (the blue module has 3 components, while the blue bundle has a single component). Another difference is the set of elements in the gray module (circled in magenta in the figure), which are data points that were unassigned to a module in WGCNA. These are part of the large green bundle in MLB, and at a later layer form a bridge set.

**Fig. 14:**
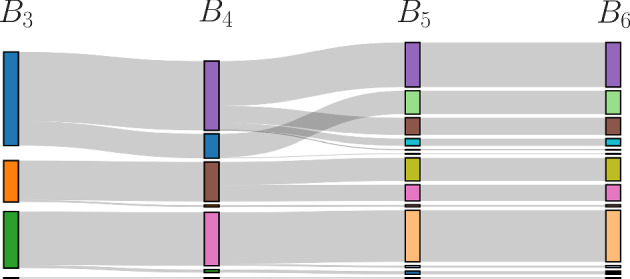
A Sankey plot of bundling layers 3 to 6 showing changes in group identity as more layers are added (Color figure online)

Both MLB and WGCNA require the user to assign parameter values. Those for WGCNA were discussed above. For MLB, the parameter is the number of layers to include. So, how do the bundles (for MLB) or modules (for WGCNA) change when parameter values are changed? That is, what is the sensitivity to parameter changes? We investigate this first with MLB. Figure 14 is a Sankey plot that shows how group membership varies from layer 3 bundles ($B_3$) to layer 6 bundles ($B_6$). The layer 3 bundles correspond to those in Fig. 13a; the length of each colored bar in the Sankey plot reflects the number of elements in the bundle. The 4 bundles in $B_3$ become 7 bundles in $B_4$, and the bundle elements transform according to the gray curves. Most elements of the blue bundle stay together in $B_4$ (in a bundle now colored purple), but some split off into a smaller blue bundle. The orange and green bundles are almost unaltered by the addition of an extra layer in the bundling process. Note that once two elements of a bundle split into separate bundles, as the layer number is increased, they can never rejoin.

**Fig. 15:**
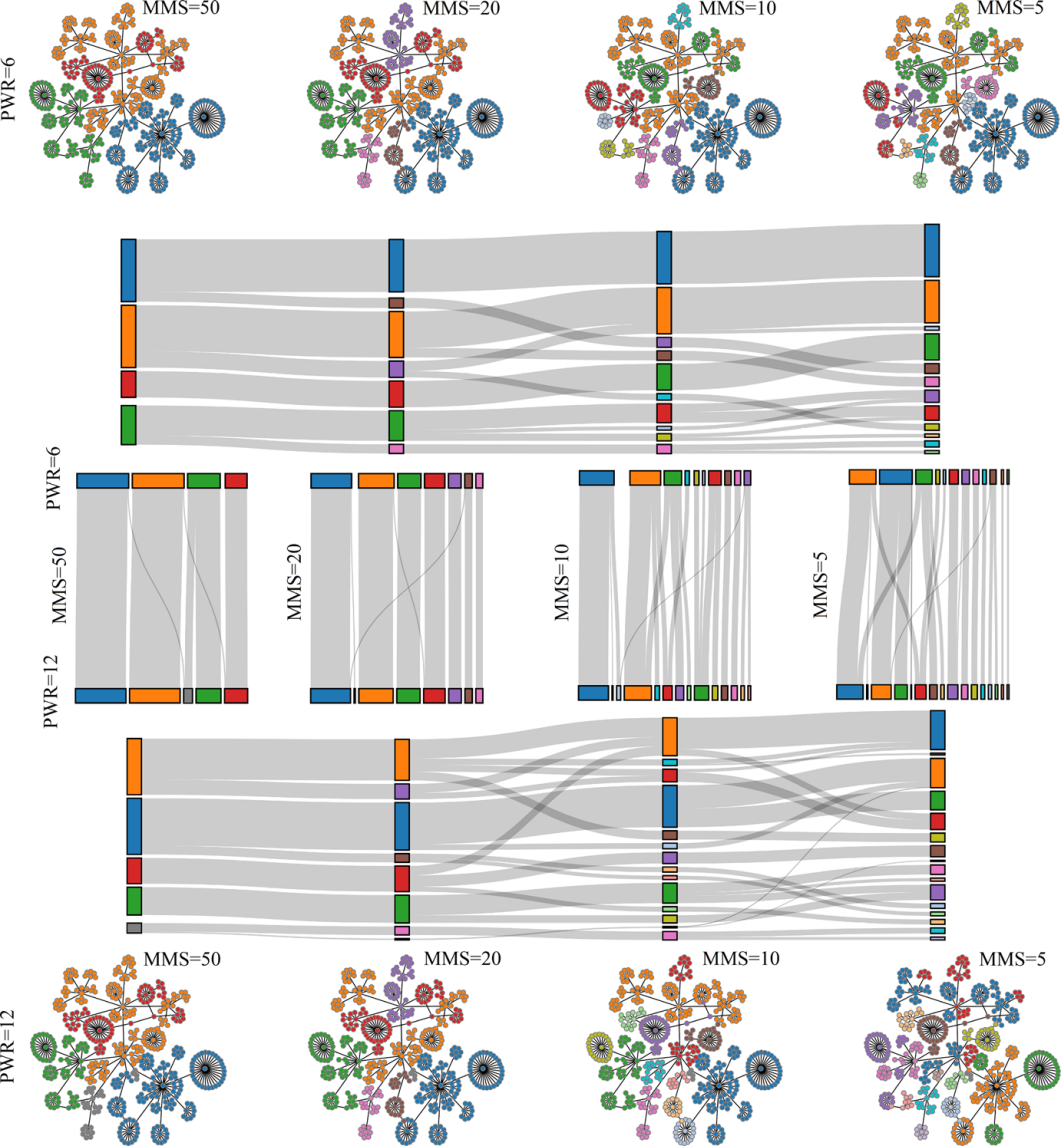
Sensitivity of WGCNA clustering to parameter changes. The networks in the upper row show modules for PWR = 6 and MMS = 50, 20, 10, and 5. The lower row of networks show modules for the same set of MMS but with PWR = 12. Sankey plots in the middle compare modules among different MMs in each row and between columns with the same MMS and different PWR. In all cases, MRT=0.2 (Color figure online)

The sensitivity analysis of WGCNA clustering is shown in Fig. 15. The top row shows how the modules change when the MMS (minimum module size) parameter is decreased from 50 down to 5, and the Sankey plot immediately below it shows the change in group membership. With MMS = 50 there are 4 modules that split into 7 modules with MMS = 20. More modules are created with smaller values of MMS, since smaller modules are allowed. However, unlike MLB, elements of a module can split into different modules as MMS is decreased, and then rejoin again as MMS is decreased further. An example of this is the orange module for MMS = 50 that splits into a large orange and a smller purple module with MMS = 20. Much of that purple module rejoins the orange module for MMS = 10. The bottom portion of the figure shows networks and a Sankey plot for a larger value of the PWR parameter (the exponent of transformation applied to elements of the similarity matrix). When the MMS parameter is now varied, one observes that, as before, the number of modules increases as MMS decreases. Also, as before, some elements of a module split into different modules, and then rejoin later for low MMS values (some elements of the blue module with MMS = 20 split at MMS = 10 and then rejoin into an orange module at MMS = 5). Finally, the Sankey plots in the middle of Fig. 15 examine changes in group identity when PWR is changed from 6 to 12, and for four different values of MMS. The most obvious change is in the number of modules, which is greater for PWR = 12 than for PWR = 6.

Overall, the sensitivity analysis indicates that in both methods the clustering varies with the choice of parameter values. However, the changes in MLB are more predictable (once elements split, they never rejoin into a common bundle), and because there are fewer parameters in MLB (just 1), the range of variation in group identity is considerably less than in WGCNA.

**Fig. 16:**
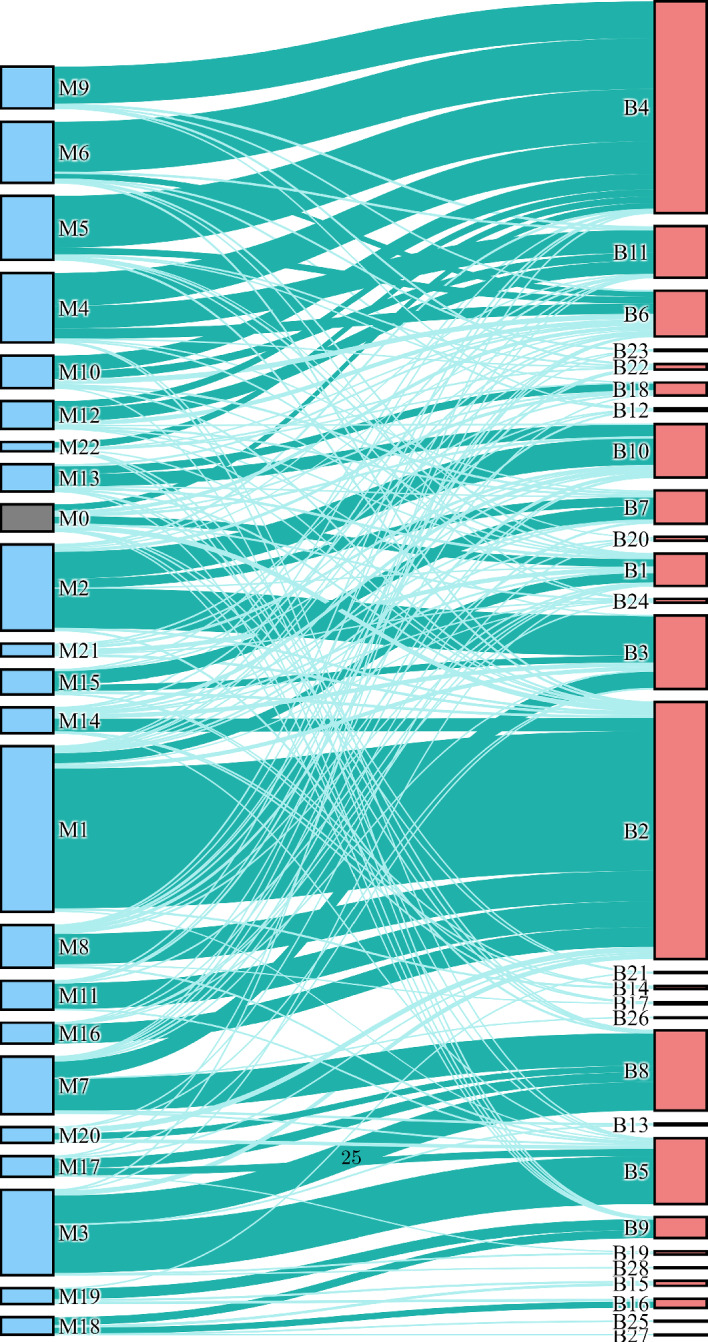
A Sankey plot of membership in WGCNA modules (with PWR = 6, MMS = 30, MRT=0) and MLB layer 4 bundles. The dark turquoise links indicate greater than 20 overlapping members between a module and a bundle, while light turquoise links indicate fewer overlapping memberships (Color figure online)

We next compare the clustering performance of WGCNA to MLB using a biological data set, publicly available data on the level of mRNA expression using a gene microarray in female mouse liver (Ghazalpour et al. 2006). It comprises 3600 of the most connected genes from 135 female mice. To find the WGCNA modules among the genes, PWR was set to 6, the lowest exponent that enforces the scale-free topology in the data similarity network, and MMS = 30 (Ghazalpour et al. 2006). In our analysis, we examined clusters obtained with MRT = 0, 0.05, 0.1, 0.15, 0.2, 0.3, and 0.4. The first column of the Sankey plot in Fig. 16 shows the resulting modules using MRT = 0 (and PWR = 6, MMS = 30). The second column shows bundles of layer 4. This illustrates the mapping of the data between modules and bundles. In some cases, genes clustered into several modules mostly lie in a single bundle (e.g., the top three modules are all contained within the top bundle), and in some cases a single module is split into several bundles (e.g., module M2 is split into bundles B3, B7, and B10).

**Fig. 17:**
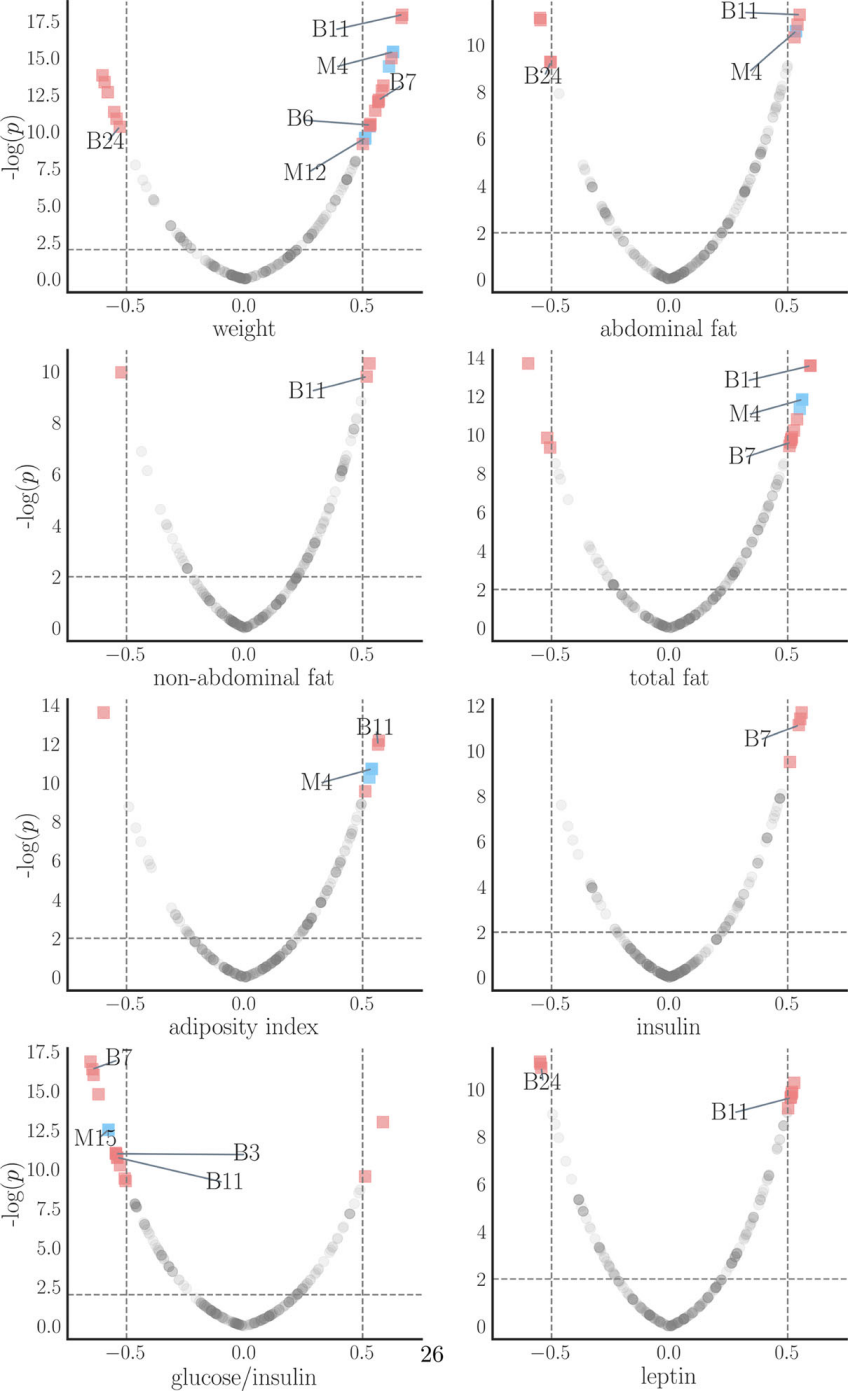
Volcano plots measuring the degree of correlation of members of modules or bundles with 8 physiological traits from a data set on gene expression in the female mouse liver. Points in the figure correspond to modules obtained using MRT =0, 0.05, 0.1, 0.15, 0.2, 0.3, or 0.4 and bundles from layers 3 to 5. Labeled points correspond to MRT=0 or bundle layer 4. Bundles with significant correlation to a trait are shown as red squares; modules with significant correlation are shown as blue squares. The gray circles indicate bundles or modules in which $|R|<0.5$ or $p<0.01$ (Color figure online)

We next tested the physiological significance of modules and bundles. To do this, we calculated the bundle/module eigengenes, the first principal component of a given bundle or module (Langfelder and Horvath 2008). This was done for all modules with MRT =0, 0.05, 0.1, 0.15, 0.2, 0.3, or 0.4 and all bundles from layers 3 to 5. Then, we evaluated the correlation coefficients (*R*) and corresponding *p*-values between bundle or module eigengenes and a list of 26 physiological traits including body weight, glucose level, and cholesterol level. For only 8 traits are there modules or bundles that are correlated with traits with $|R|>0.5$ and $p<0.01$. Figure 17 show volcano plots for these eight traits, where each data point corresponds to a module or bundle. The red squares indicate bundles with significant correlation with a trait, while the blue squares indicate modules with significant correlation. Labels correspond to modules with MRT=0 or layer 4 bundles. For each trait, the cluster with the highest correlation coefficient (in absolute value) corresponds to a bundle. For some traits, non-abdominal fat, insulin, and leptin, the only clusters with $|R|>0.5$ are bundles. This analysis indicates that, for this data set and choice of parameters, MLB outperforms WGCNA with regard to the correlation of cluster membership with physiological traits.

## Discussion

Spectral clustering is an often-used method for grouping data. This comes with uncertainty, however, in knowing which clustering regime to use. The MLB method bypasses this by using the several most prominent clustering regimes to determine bundles, each of which provides a view of the data from global to local as one moves into deeper bundling layers. The most appropriate number of layers to use can be inferred from an examination of the bundle sizes at different layers (Fig. 5). The bundling method also identifies bridge sets in the data that serve as links between bigger bundles. These bridge sets, which are transitional data coupling two larger data modules, are less discernible using spectral clustering alone. The iterative bundling process also provides information that can be used to determine the relationship between different bundles. Thus, the method not only forms multi-layer groupings of the data, but also connects these groupings based on how they are bundled together over different layers.

MLB inherits the data dimensionality reduction power of spectral clustering but does it in a systematic and iterative approach. It does this using the raw affinity data, without modification. This is in contrast with the popular software package WGCNA (Zhang and Horvath 2005; Langfelder and Horvath 2008, 2012; Zhao et al. 2010; Van Dam et al. 2017), which converts the affinity matrix into an adjacency matrix by raising each element to a power (a soft-thresholding parameter, PWR) such that the degree distribution of the corresponding network follows a power law distribution. The resulting adjacency matrix is then transformed into a Topological Overlap Matrix (TOM). This is a form of averaging that can weaken the association between two elements of the network. Finally, WGCNA applies hierarchical clustering to the TOM to cluster genes into modules, and the number of modules is determined by the user by setting thresholds (MMS,MRT). Bundling, in contrast, makes direct use of the affinity data, and provides the number of bundles (clusters) for each layer without user input.

The bridge bundles that can be identified with bundling are similar in some ways to nodes that have been referred to as articulation points or cut vertices in networks, whose removal increases the number of connected components in the network (Tian et al. 2017). The Depth-First Search (DFS) algorithm can be used to find such special nodes in a network (Even 2011; Tarjan and Vishkin 1985). Also, centrality measures such as betweenness centrality (Freeman 1977; Newman 2018; Freeman 1978), which identifies nodes that act as bridges, and percolation centrality (Piraveenan et al. 2013), which identifies nodes that are crucial for maintaining the connectedness of the network, are used to identify bridge nodes. These methods all work on networks, where MLB works with data sets. They also differ from our approach to finding bridge sets in that they do not take into consideration the size of the clusters. Why does the size of a cluster or bundle matter in the identification of bridge sets? We view a large cluster/bundle as a collection of data with extensive similarities that share a categorical identity. For example, a large cluster/bundle could reflect upregulation of a set of genes related to inflammation in a particular disease state. Two large clusters/bundles would then reflect two distinct categorical states. In contrast, we view a bridge set as transitional between these, with similarities to both, but not as a separate categorical state. For this reason, we define bridge sets to have small cardinality relative to the clusters or bundles they join together.

The network reconstruction that we performed was an effort to use bundle information to identify the structure of the network that gave rise to positive and negative correlations in the data. The algorithm for forming the L-bundle and H-bundle networks is only one possible method for converting the information in the L and H matrices into network structures. Improvements to this algorithm could yield better reconstructions of the bundle network, and point to a direction of future potential development. One major advantage of using synthetic data in which correlations in data are based on a structural network, as we did here, is that it is possible to compare the reconstituted bundle network to a ground truth bundle network. In real life applications, this will not be possible, but the reconstruction process will instead be a very useful tool for establishing relationships between the bundles, even if there are false positives and negatives in the edges in the reconstituted network. Bundles that are highly related, i.e., neighbors in the reconstructed bundle network, may reflect sets of genes whose gene products are elements of pathways impacted by a disease, for example. One advantage of the iterative bundling process, then, is that not only are the most highly correlated elements of the network grouped together into bundles, but the history of clustering is not lost and can be recapitulated in the reconstituted bundle network.

In the final section of the manuscript we compared the performance of MLB to that of WGCNA using both synthetic and biological data sets. The comparison with synthetic data indicates a strong dependency of WGCNA clustering on the choice of parameter values, while one advantage of MLB is that there is a single parameter (the number of layers to use). The comparison with the biological data set indicated superior performance of MLB over WGCNA with regard to correlation with physiological traits, but it is possible that with other parameter choices WGCNA would have exhibited better performance. If so, then this again shows the importance of choosing good parameter values with WGCNA, where in most cases there will be little basis for making such a choice.

## Data Availability

Not applicable.

## Code Availability

On demand.
